# Individual and interactive effects of warming and nitrogen supply on CO_2_ fluxes and carbon allocation in subarctic grassland

**DOI:** 10.1111/gcb.16851

**Published:** 2023-07-10

**Authors:** Kathiravan Meeran, Niel Verbrigghe, Johannes Ingrisch, Lucia Fuchslueger, Lena Müller, Páll Sigurðsson, Bjarni D. Sigurdsson, Herbert Wachter, Margarete Watzka, Jennifer L. Soong, Sara Vicca, Ivan A. Janssens, Michael Bahn

**Affiliations:** ^1^ Department of Ecology University of Innsbruck Innsbruck Austria; ^2^ Research Group Plants and Ecosystems University of Antwerp Antwerp Belgium; ^3^ Centre for Microbiology and Environmental Systems Science University of Vienna Vienna Austria; ^4^ Agricultural University of Iceland Borgarnes Iceland; ^5^ Soil and Crop Sciences Department Colorado State University Fort Collins Colorado USA

**Keywords:** ^13^CO_2_ pulse labeling, carbon allocation, gross primary productivity, nitrogen addition, soil respiration, soil warming

## Abstract

Climate warming has been suggested to impact high latitude grasslands severely, potentially causing considerable carbon (C) losses from soil. Warming can also stimulate nitrogen (N) turnover, but it is largely unclear whether and how altered N availability impacts belowground C dynamics. Even less is known about the individual and interactive effects of warming and N availability on the fate of recently photosynthesized C in soil. On a 10‐year geothermal warming gradient in Iceland, we studied the effects of soil warming and N addition on CO_2_ fluxes and the fate of recently photosynthesized C through CO_2_ flux measurements and a ^13^CO_2_ pulse‐labeling experiment. Under warming, ecosystem respiration exceeded maximum gross primary productivity, causing increased net CO_2_ emissions. N addition treatments revealed that, surprisingly, the plants in the warmed soil were N limited, which constrained primary productivity and decreased recently assimilated C in shoots and roots. In soil, microbes were increasingly C limited under warming and increased microbial uptake of recent C. Soil respiration was increased by warming and was fueled by increased belowground inputs and turnover of recently photosynthesized C. Our findings suggest that a decade of warming seemed to have induced a N limitation in plants and a C limitation by soil microbes. This caused a decrease in net ecosystem CO_2_ uptake and accelerated the respiratory release of photosynthesized C, which decreased the C sequestration potential of the grassland. Our study highlights the importance of belowground C allocation and C‐N interactions in the C dynamics of subarctic ecosystems in a warmer world.

## Graphical abstract

Climate warming has been suggested to impact high latitude ecosystems, potentially causing significant carbon (C) losses from soil, while also stimulating nitrogen (N) turnover. Conducting a ^13^C pulse labeling experiment on a 10‐year geothermal warming gradient in subarctic grassland (Forhot.is) we tested for the effects of soil warming and experimental N addition on CO_2_ fluxes and the fate of recently photosynthesized C. Our findings indicate that the unexpected warming‐induced limitations in N availability for plants and C availability for soil microbes resulted in a decrease in the grassland's net ecosystem CO_2_ uptake and accelerated the release of recently photosynthesized C.

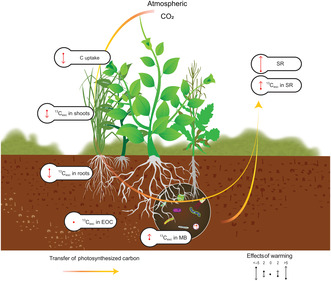

## 1 INTRODUCTION

The Earth's warming is most pronounced in the high latitudes (IPCC, 2021). Northern ecosystems contain more carbon (C) in soil than the atmosphere (Ping et al., 2008) and the fate of this (long‐term) stored C under changing climate conditions is important and highly debated (Crowther et al., 2016; van Gestel et al., 2018). Experimental and modeling studies suggest that Northern ecosystems will release substantial amounts of C with soil warming (Koven et al., 2017; McGuire et al., 2009; Verbrigghe, Leblans, et al. 2022). Soil warming can also increase N mineralization rates (Bai et al., 2013; Rustad, 2008). The resulting increase in N availability can increase gross primary productivity (GPP) and plant growth (Zhou et al., 2022), which could potentially compensate for the warming‐induced loss of soil C (Chen et al., 2015; Melillo et al., 2011; Zhang et al., 2020). Our current understanding of the consequences of warming on the major components of the C cycle often lacks explicit consideration of direct versus indirect (e.g., N availability‐related) effects of warming and is largely based on short‐term experiments (Song et al., 2019), which may not adequately account for temporal shifts in acclimation responses, substrate availability and belowground communities (Domeignoz‐Horta et al., 2023; Melillo et al., 2017; Romero‐Olivares et al., 2017). To date, there is a major knowledge gap on whether and how the two largest terrestrial fluxes of CO_2_, that is, GPP and soil respiration (SR), and their coupling through the process of belowground C allocation (Hartmann et al., 2020) are affected by the interaction of sustained warming and N availability in Northern ecosystems.

Plants allocate a significant fraction of the C assimilated from the atmosphere through photosynthesis (GPP) belowground, where it is invested into root biomass, to metabolic activity and respiration or is released as exudates, incorporated into microbial biomass (MB) and used as energy source for respiration (Brüggemann et al., 2011). It is being increasingly recognized that plant C allocation is strongly driven by the C demand of sink organs (Fatichi et al., 2019; Körner, 2015), which in turn can respond to the environmental conditions such as temperature, water, and nutrient availability (Hasibeder et al., 2015; Sarker et al., 2017). Soil warming has been shown to alter belowground C allocation, with some studies reporting an increased allocation (Yin et al., 2013; Zong et al., 2018), while others report a decreased belowground allocation (Bai et al., 2010; Xiong et al., 2020). This inconsistency could be due to altered N availability induced by warming (Wang, Chen, et al., 2021; Wang, Defrenne, et al., 2021). Warming‐induced N mineralization can increase the availability of N and promote plant growth (Natali et al., 2012). At the same time, high N availability can also lead to a decrease in C allocation to rhizosphere microbes and soil (Sun et al., 2019; Xiao et al., 2019), as the need for belowground investment for N through root growth and exudation decreases. The indirect effects of warming on belowground carbon allocation through altered N availability are still poorly understood. However, improving our understanding of these effects is crucial to accurately predict future carbon dynamics and inform Earth system models (Bouskill et al., 2014; Chadburn et al., 2017; Schädel et al., 2018).

The rapid transfer of recent C from photosynthesis to SR links the two largest fluxes of CO_2_ in terrestrial ecosystems and strongly contributes to the magnitude (up to 60%) and diel‐dynamics of SR (Bahn et al., 2009; Kuzyakov & Gavrichkova, 2010). Environmental changes can strongly alter this coupling of photosynthesis and SR (Blessing et al., 2016; Ingrisch et al., 2020; Meeran et al., 2021). Next to photosynthetic C supply to SR, the turnover of the photosynthesized C determines the rate of release of C to the atmosphere. Soil warming, on the one hand, can directly increase process‐rates and increase the C demand for root (Järvi & Burton, 2020) and microbial metabolism (Hartley et al., 2007; Walker et al., 2018), leading to increased turnover of belowground C and SR (Reinthaler et al., 2021; Wan et al., 2007). The increased belowground turnover can lead to loss of C from the ecosystem through priming of soil organic matter (Hartley et al., 2012). On the other hand, N addition may lead to increased allocation of C to plant structures, slowing down the turnover of photosynthesized C and increasing its residence time (Xiao et al., 2019). However, the interaction between warming and N availability on C turnover and SR, as well as the response of photosynthetic control on SR, remain poorly understood. This knowledge gap contributes significantly to the uncertainty of global vegetation models (Friend et al., 2014).

In this study, we investigated the effects of 10 years of soil warming and N availability on major ecosystem CO_2_ fluxes, plant C allocation to above‐ and belowground biomass, and microbial incorporation of plant‐derived C, as well as the coupling of photosynthesis and SR. We used a natural geothermal warming gradient (0–8.7°C) (Sigurdsson et al., 2016) to study the effects of warming on recently photosynthesized carbon in a subarctic ecosystem that contains significant carbon stocks and experiences the full range of projected warming for high latitudes (IPCC, 2021; Soong, Phillips, et al., 2020). We added N (in the form of NH_4_NO_3_) to half of the study‐plots along the warming gradient to examine the interacting effects of warming and N addition. We hypothesized that (H1) warming and N addition, both individually and combined, would stimulate ecosystem productivity, as increased N mineralization under warming as well as N addition should increase N availability to plants, and that (H2) warming and N addition would decrease belowground allocation as a consequence of increased N availability. Additionally, we hypothesized that (H3) warming would increase the turnover of recently assimilated C and SR due to increased allocation to metabolic activity; we also expected that N addition would increase C allocation to plant structure rather than respiration. Therefore, when combining the two factors, N addition would diminish the warming effects on C turnover and SR.

## 2 METHODS

### 2.1 Experimental site and design

The study was performed in a natural soil warming gradient located at Reykir, Iceland (64.008° N, 21.178° W) and is part of the “ForHot” experimental infrastructure (Sigurdsson et al., 2016). The soil at the site is classified as a Brown Andosol (Arnalds, 2015). The mean annual air temperature of the site is 5.2°C, and the mean annual precipitation is 1457 mm (Sigurdsson et al., 2016). The soil warming gradient was formed in May 2008, when a major earthquake affected a geothermal system and shifted it to a previously unwarmed surface. The intensity of this temperature gradient did not change, and the warming was found to be stable over time (Sigurdsson et al., 2016). This then newly warmed soil is covered by unmanaged treeless grassland dominated by *Agrostis capillaris*, *Poa pratensis, Ranunculus acris* and *Equisetum pratense*. The plots for this experiment were established in June 2017 on two soil warming transects, with each plot measuring 2 × 2 meters in size. The plots were randomly installed along the warming gradient. The species composition of the grasslands was not different between the studied plots. The warming levels, or the difference in soil temperature compared to unwarmed plots, ranged from 0 to 8.7°C (Figure S2). The warming levels encompass the full range of warming projected for Northern ecosystems (up to +6.4°C) by the year 2100 under Representative Concentration Pathway 8.5 (IPCC, 2021). The present study utilized a total of 14 plots. To study the interactive effects of warming and N addition, half the number of plots (seven) were fertilized twice a year (May and August) with solid NH_4_NO_3_ (50 kg N ha^−1^ year^−1^) starting in 2017. The N addition plots were chosen to have similar soil warming conditions as the plots without N addition. The warming for each unfertilized plot was found to be 0, 0.5, 1.5, 6.1, 6.6, 7.7, and 8.7°C, respectively. In the N addition plots, warming ranged from 0.3 to 8.1°C, with values of 0.3, 0.8, 1.5, 5.1, 6.5, 7, and 8.1°C above ambient. While the initial state of soils is an important factor to consider in experimental warming studies, the unpredictable nature of the earthquake‐triggered warming precluded obtaining any data on the pre‐warmed soil state. We therefore assumed that the control plots without warming represent the ecosystem state and processes prior to the earthquake event.

During July 2018, the photosynthetically active radiation (PAR; S‐LIA‐M003; Onset Computer Corporation) and air temperature (sensors S‐TMB, logger HOBO Micro Station H21‐002; Onset Computer Corporation) at 1 m height were recorded at the study site (Figure S1). In each plot, soil temperature (Figure S2) and volumetric soil water content (SWC: Figure S3) were continuously measured at 5 cm soil depth (S‐TMB and 10HS, HOBO Micro Station H21‐002; Onset computer corporation).

### 2.2 ^13^CO_2_ pulse labeling

Pulse labeling with ^13^CO_2_ was performed under clear sky conditions on two consecutive days (July 16 and 17, 2018). The protocol was similar to previous studies (Bahn et al., 2013; Ingrisch et al., 2020; Meeran et al., 2021). Four days before pulse labeling, plastic frames (50 × 50 cm) were installed in all 14 plots. For the pulse labelling, a plexiglass chamber (50 × 50 × 50 cm) was placed on top of the plastic frames. Rubber gaskets were used between the chamber and the frames to avoid gas leakages. The chambers were ventilated and temperature was controlled using fans and circulating cold water with 6 mm diameter tubes inside the chamber. Air temperature, CO_2_ concentration inside the chamber, and PAR outside the chamber were continuously monitored during labeling. The temperature inside the chamber was in the range of 20 ± 5°C. The isotopic ratio (^13^C/^12^C) was measured using an online isotope laser (Picarro G2201i Analyzer; Picarro Inc). Before labelling, the CO_2_ concentration inside the chamber was reduced to below 250 ppm by plant photosynthesis and by scrubbing using soda‐lime. Then highly enriched (>99%) ^13^CO_2_ was added as 10–15 mL pulses to achieve 40–60 atom‐% ^13^C and to maintain CO_2_ concentration below 800 ppm. Each labeling lasted for 60 ± 10 min.

### 2.3 Plant and soil sampling

Plant and soil samples were collected at each plot immediately after pulse labeling was completed, and then after 1, 3, 6 and 10 days. Natural abundance samples were collected from each of the plots 1 day before labeling. For shoot sampling, a ring (Ø = 5 cm) was placed on the soil, after which all the shoot biomass within the ring was clipped to the ground. The metabolic activity of freshly collected shoots was immediately stopped by freezing in liquid nitrogen. Soil samples from the upper 7 cm were taken directly below the cut surface, using a soil auger with an inner diameter of 5 cm. The soil samples were immediately sieved to 2 mm. Aliquots of fresh soil were dried at 70°C for 38 h. Roots were washed from soil and filtered for dead roots, and coarse roots (diameter >2 mm). The C and N concentration and isotope ratio of the shoot, root, and soil samples were analysed using elemental analysis (EA)‐ IRMS (EA 1100, CE Elantech; coupled to a Delta+ IRMS; Finnigan MAT).

In subsamples of fresh soil, the MB C and N were measured on the day of sampling following the chloroform fumigation extraction method (Vance et al., 1987). Briefly, 2 g of the chloroform fumigated (for 24 h) and non‐fumigated soil aliquots were extracted with 20 mL of 0.5 M K_2_SO_4_. The extracts were then analyzed for extractable organic carbon (EOC) and total extractable nitrogen (TEN) using a TOC analyzer (TOC‐V CPH E200V/TNM‐122V; Shimadzu). The difference of EOC and TEN between fumigated and non‐fumigated extracts were considered as the MB C and N. The δ^13^C of MB C and EOC in fumigated and non‐fumigated extracts was measured using liquid chromatography (Dionex Corporation) coupled to an isotope ratio mass spectrometer (IRMS Finnigan MAT). In non‐fumigated K_2_SO_4_ extracts ${\mathrm{NO}}_{3}^{-}$ and ${\mathrm{NH}}_{4}^{+}$ concentrations were determined using colorimetric methods (Hood‐Nowotny et al., 2010).

### 2.4 Normalized difference vegetation index measurements

NDVI (normalized difference vegetation index) was measured using a handheld SpectroSense 2+ four‐channel sensor (Skye Instruments). The measurements were made on a fixed location in each plot by placing the sensor pole in a premarked corner of the plot and tilting the pole (approximately 74°) in the direction of the opposite diagonal corner. The measurements were made at a height of 2 m covering a measurement surface of 0.62 m^2^. The calculation of NDVI was done as described by (Tucker, 1979) and the following equation:

(1) NDVI=34𝜌840−34𝜌660/34𝜌𝜌840+34𝜌660.

NDVI was measured for the 14 plots under study during the growing season (April–September) of 2018. The limited accessibility and adverse weather conditions at the study site limited the opportunities for NDVI measurements. Nevertheless, reproducible NDVI measurements could be taken on a total of 9 days between 9 AM and 4 PM across the growing season.

### 2.5 CO_2_ flux measurements

Gross primary productivity was calculated from net ecosystem exchange (NEE) and ecosystem respiration (ER), both of which were measured using the same plexiglass chamber as was used for pulse labeling. The procedure was similar as described in Schmitt et al. (2010) and Ingrisch et al. (2018). Briefly, the transparent plexiglass chamber was placed on the plastic frame and concentrations of CO_2_ and water vapour, as well as air temperature, was monitored at 5‐s intervals for 1 min (GMP 343, Vaisala; HMP 75, Vaisala). During the NEE measurements, PAR (PQS1 PAR Quantum Sensor; Kipp & Zonen) was recorded. The ER measurements were conducted by covering the chamber with a dark cloth. Measurements were quality controlled visually (Pirk et al., 2016) and the CO_2_ flux rates were calculated by linear regression, as described by similar studies (Ingrisch et al., 2018; Schmitt et al., 2010). The NEE and ER measurements were conducted between July 8 and August 4, 2018, with randomized order of plots. GPP was calculated as the difference of NEE and ER. To ensure comparability and visualize treatment effects, we present the light‐saturated GPP (GPP_max_; at PAR > 1000 μmol m^−2^ s^−1^). We use the convention that positive NEE values represent a net ecosystem CO_2_ source, and negative NEE values represent a net CO_2_ sink.

Soil respiration and its isotopic composition were measured by two methods. For the eight plots in the transect with access to mains power supply, SR and its isotopic composition were continuously measured using a custom made steady‐state measurement setup as described by (Ingrisch et al., 2020; Meeran et al., 2021). Briefly, the measurement was conducted using a PVC chamber with 4.5 cm diameter and 10 cm height. Each chamber had two connections one of which was connected to a buffer volume to stabilize the concentration of CO_2_ entering the chamber. The other end was connected to an online isotope analyzer (Picarro G2201i Analyzer; Picarro Inc) through a valve multiplexing system. For each SR measurement, the concentration of isotopologues of CO_2_ (^12^CO_2_ and ^13^CO_2_) inside the buffer volume (for 6 min) and air from the chamber (for 8 min) were alternatively measured. SR was calculated as

(2) $$\mathrm{SR}=\frac{{\mathrm{CO}}_{2\left(\text{chamber}\right)}-{\mathrm{CO}}_{2\left(\text{buffer}\right)}}{\text{Area of chamber}}\times \text{flowrate}.$$

The ^13^C atom fraction of SR 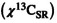 was calculated as
(3)

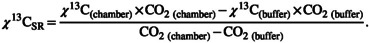




The isotope analyzer was calibrated using two calibration gases (430 and 2926 ppm) at the end of each measurement cycle. The isotopic composition of the calibration gases (−7.6‰ and −3.7‰) were measured using gasbench‐IRMS (Finnigan MAT).

For the remaining six more remote plots without electrical power supply, collars made of PVC tubes with 10 cm diameter were installed into the ground/soil and the vegetation inside was removed. SR was measured manually using a portable infra‐red gas analyzer (EGM‐4; PP Systems). The isotopic composition was measured by accumulating soil‐respired CO_2_ for 30 min and sampling 10 mL of gas inside the chamber 1, 3, 5, 15, and 30 min after closing the chamber. The gas samples were analyzed on a gasbench‐IRMS (Finnigan MAT). The isotopic composition of SR was calculated using the Keeling plot approach (Drake et al., 2019; Keeling, 1961). To ensure comparability between the two measurement methods, non‐steady‐state measurements (approach 2) were also made on the plots with steady state measurements (approach 1). The range of SR was similar between methods, and the treatment effects were preserved (Figure S4).

The ForHot study area is prone to geogenic CO_2_ efflux along the geothermal soil warming gradients (Maljanen et al., 2020). The contribution of geogenic CO_2_ efflux (Figure S5) was calculated using a two‐pool mixing model with isotopic composition of geogenic source as −4.7‰ and biogenic source as −28‰ (Maljanen et al., 2020). In the studied plots, the amount of geogenic CO_2_ efflux was not correlated to soil warming (Figure S5). The SR values reported were corrected for geogenic CO_2_ efflux.

### 2.6 Data analysis and statistics

The absolute amount of label ^13^C recovered (excess ^13^C) in shoot, root, MB, EOC, and SR were calculated as
(4)

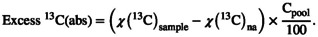

Here, 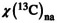 and 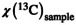 are the ^13^C atom fractions measured in the samples before and after labeling, respectively. C_pool_ represents the amount of C in shoot, root, MB, EOC, and SR.

The ^13^C excess in shoot biomass measured immediately after labeling was considered to correspond to the amount ^13^C incorporated during ^13^CO_2_ pulse labeling. For each plant and soil C pool, the relative amount of ^13^C recovered was calculated using ^13^C incorporated into shoots immediately after labeling as total ^13^C taken up,
(5)

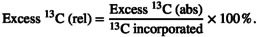




The continuous measurements (in eight plots) of soil‐respired CO_2_ and ^13^CO_2 exc_ (^13^CO_2_ excess) were evaluated for temporal lags from the environmental drivers as described by Meeran et al. (2021). Briefly, the time lags between the diel dynamic of environmental drivers (PAR, soil temperature, SWC) and SR variables (CO_2_ and ^13^CO_2 exc_) were evaluated by stepwise shifts (at intervals of 2.4 h) in the time‐series (spanning ±20 h) of SR followed by modelling the effects of drivers (PAR, soil temperature, SWC) on SR. The most probable time lag between the dynamics of SR and the environmental driver is the time shift at which the coefficient was the highest. Negative time‐shifts indicate that the driver leads before the response variable. Positive time‐shifts indicate that the driver did not affect the dynamics of the response variable. The regression coefficients from the model for each plot was computed and grouped (*n* = 2) according to warming level (low: 0–1.5°C; high: 5.1–8.1°C) for improved visualization and testing treatment effects. Because of less sample sizes (*n* = 8 time‐lag estimates), the individual effects of warming and N addition on time‐lags were tested using permutational ANOVA (R‐package “lmPerm”; Wheeler & Torchiano, 2016).

The rate of decrease of ^13^C_exc_ in a component can be described by an exponential function,

(6) $$y=Ae^{-\mathrm{bt}},$$

where *A* is ^13^C_exc_ at the peak time, *b* is the decay constant and *t* is time from labeling. To this end, an exponential model was fitted for ^13^C_exc_ in shoot and SR (as a proxy for decrease in respiratory substrates), using the R‐function ‘nls’. The mean residence time (MRT) of ^13^C_exc_ was calculated as

(7) MRT=1/b.

Immediately after pulse labeling, the physical back‐diffusion of ^13^CO_2_ tracer from soil can increase ^13^C_exc_ in soil CO_2_ efflux. Previous studies (Ingrisch et al., 2020; Meeran et al., 2021) using same pulse‐labeling techniques have found that the proportion of diffused tracer was minimal (ca. 4%) with rapid turnover (ca. 25 min) compared to the respired ^13^CO_2_. Hence, in this study, the MRT of ^13^C_exc_ in SR was calculated by excluding data from first measurement cycle (2.4 h) after labeling.

Multivariate analysis was performed in the form of structural equation modelling using ‘piecewise SEM’ (Lefcheck, 2016) to unravel the direct effects of warming on C allocation and indirect effects through altered N availability. The pathways of the piecewise SEM were fitted as linear mixed‐effects models with the days since labelling considered as a random factor. Because of collinearity between C and N contents in microbes, MB was calculated as the mean of standardized microbial C and N. The model was built including all hypothetical pathways testing the direct and indirect effects of warming, plus the effect of N addition and its interaction with each pathway (Figure S6). Tested pathways that were statistically non‐significant (*p* > .05) and generated a high Akaike information criterion score (AIC > 300), were excluded from the model and the model was further optimized to account for more variation. The final optimized model was selected based on the lowest AIC score, and chi‐square statistics were run to evaluate the model goodness‐of‐fit (Shipley, 2009). If the chi‐square was statistically non‐significant (*p* > .05) the model was a good fit to the data. The Fisher's *C* and *p* value for the final optimized SEM were 92.4 and 0.2, respectively.

To analyze the effects of warming, N addition and their interaction on belowground C allocation and CO_2_ fluxes, we used linear mixed effects models with the R‐package “nlme” (Pinheiro et al., 2021). The model was formulated as follows:

(8) $$\begin{array}{c} \mathrm{lme}\left(\text{response}∼\text{warming}\;\times \;N-\text{addition},\text{random}\;=\;∼1|\text{date},\text{method}\right.\\ \;=\text{‘REML}’,\left.\text{data},\;,=,\;,\text{data}\right). \end{array}$$

The response variables in our models were CO_2_ fluxes, NDVI and the amount (both absolute and relative) of recent C in shoots, roots, EOC, microbes, and SR. The fixed effects comprised the main effects of warming and N addition, as well as their interaction. Soil warming was treated as a continuous variable, considering the gradient ranging from 0 to 8.7°C. N addition was included as a factor variable, with two levels (0 and 50 kg/ha). The random effects in our models included the days of sampling as random intercepts. We used restricted maximum likelihood estimation to estimate the fixed and random effects coefficients in our models and performed likelihood ratio tests to assess the significance of the effects. The models were considered to be significant if the *p*‐value for the likelihood ratio test was less than .05. The NDVI measurements were grouped for early (days until NDVI saturation levels), peak (NDVI at saturation levels) and late (start of decline in NDVI) season, and the models were performed for each seasons. To meet the assumptions of normality in linear mixed effects modeling, we assessed the normality of model residuals using Shapiro and Wilk's statistic (Royston, 1995). The skewed response variables were ^13^C_exc_ in leaf, root, MB, EOC, and SR, as well as NDVI, GPP, NEE, and ER. These variables were log‐transformed prior to model fitting. All statistical analysis were performed in R (R Core Team, 2022).

## 3 RESULTS

### 3.1 NDVI and ecosystem and soil CO_2_ fluxes

**FIGURE 1:**
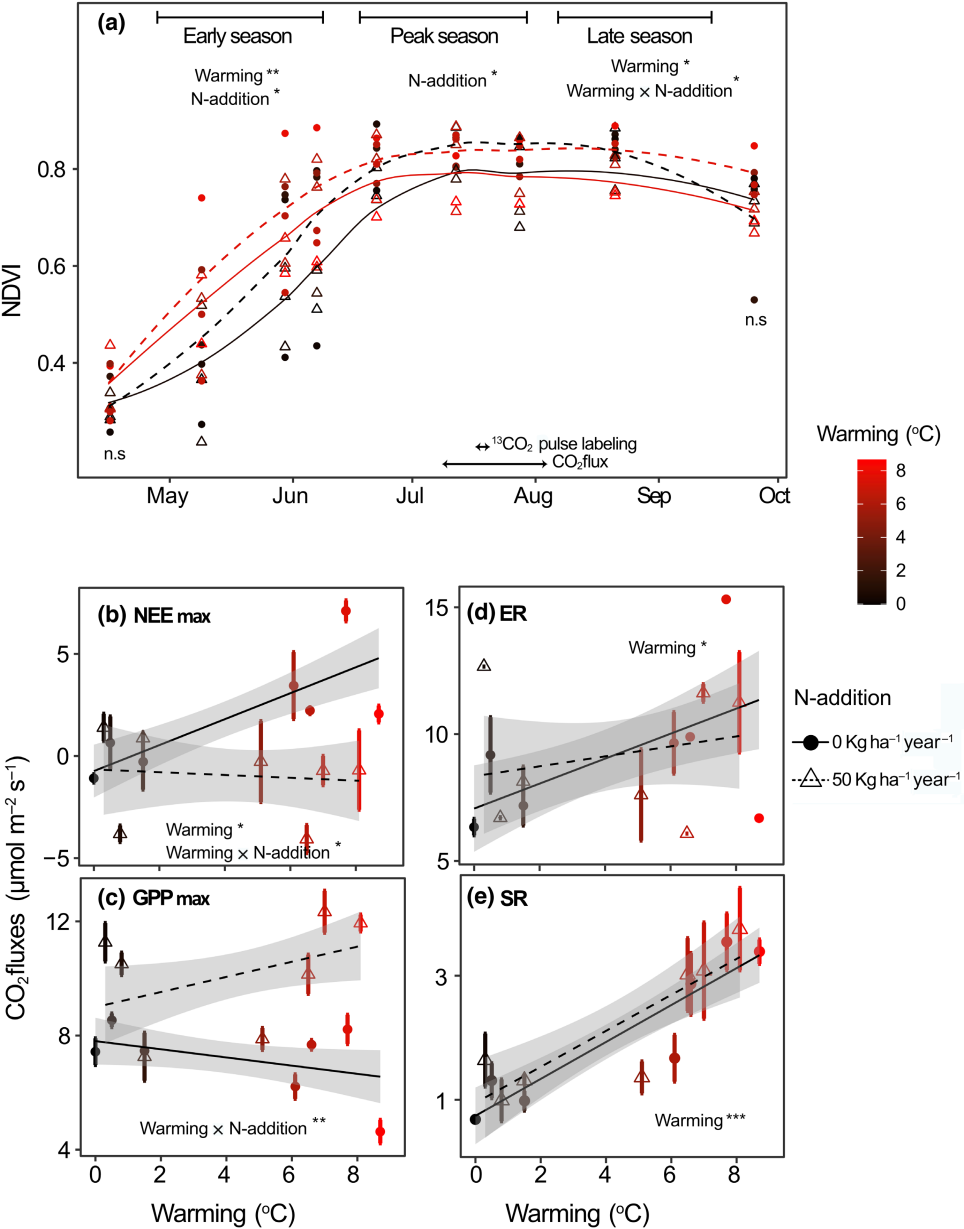
Normalized difference vegetation index (NDVI) and ecosystem CO_2_ fluxes in response to warming and N addition. (a) NDVI measured during the early, peak, and late growing season. (b) Light saturated (PAR > 1000 μmol m^−2^ s^−1^) net ecosystem exchange of CO_2_ (NEE_max_) and (c) gross primary productivity (GPP_max_), (d) ecosystem respiration (ER) and (e) soil respiration (SR) measured along the soil warming gradient in July 2018. The ambient soil temperature of the unwarmed plots during the field campaign was 10.2°C. Circles and triangles indicate measurements from unfertilized and fertilized (N‐addition) plots, respectively. Error bars indicate standard errors of the mean. Asterisks denote significant effects of warming, N‐addition and their interaction (*p* value: <.05*; < .01**; <.001***).

Warming and N addition significantly increased NDVI during the early season (Figure 1a; warming: *t*‐value = 3.5, df = 37, *p* < .01; N addition: *t*‐value = 2.0, df = 37, *p* < .05). However, the interaction between warming and N addition did not show a significant effect on NDVI during the early season. During peak growing season, when the pulse labeling and CO_2_ flux measurements were performed, NDVI of all plots was saturated and N addition increased NDVI significantly (*t*‐value = 3.3, df = 37, *p* < .05), while no significant effects were observed for warming alone or the interaction between warming and N addition. At the end of the growing season, NDVI was decreased by warming (*t*‐value = −1.5, df = 23, *p* < .05), and increased by interaction of warming and N addition (*t*‐value = 2.1, df = 23, *p* < .05). Overall, warming and N addition increased NDVI significantly during the growing season (*p* = <.05), the effect being significant for most individual dates, except for the very beginning and ending of the field season (first and last measurement date, respectively). Warming increased NEE_max_ (NEE at light saturation; Figure 1b; *t*‐value = 3.3, df = 51, *p* < .05), but did not affect GPP_max_ (GPP at light saturation; Figure 1c). Warming increased both ER (Figure 1d; *t*‐value = 2.4, df = 51, *p* < .05) and SR (Figure 1e; *t*‐value = 8, df = 63, *p* < .001). The warming and N addition in combination significantly increased GPP_max_ and decreased NEE_max_ (Figure 1b,c; GPP_max_: *t*‐value = 2.8, df = 51, *p* < .01; NEE_max_: *t*‐value = −2.76, df = 51, *p* < .05) but did not affect ER and SR (Figure 1d,e).

### 3.2 ^13^C tracer in plant, soil and SR

**FIGURE 2:**
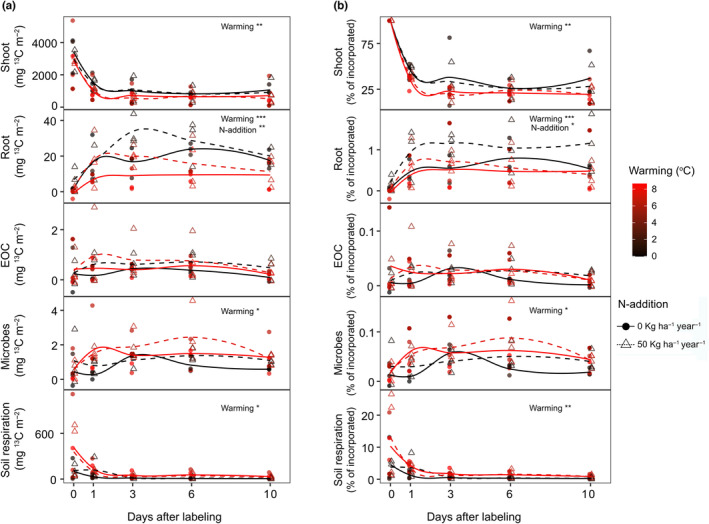
Dynamics of the (a) absolute and (b) relative amount of ^13^C excess in shoots, roots, extractable organic carbon (EOC), microbes, and soil respiration measured after ^13^CO_2_ pulse labeling. The relative amounts of ^13^C excess were calculated as percentage of ^13^C excess relative to ^13^C excess in shoots immediately after labeling. Circles and triangles indicate measurements from unfertilized and fertilized (N‐addition) plots, respectively. Smoother lines indicate mean variation of ^13^C excess grouped based on soil warming levels (Black: 0–1.5°C, Red: 5.1–8.1°C) and N‐addition treatments (dashed lines). Asterisks denote significant warming and N addition treatment effects (*p* value: <.05*; <.01**; <.001***).

**FIGURE 3:**
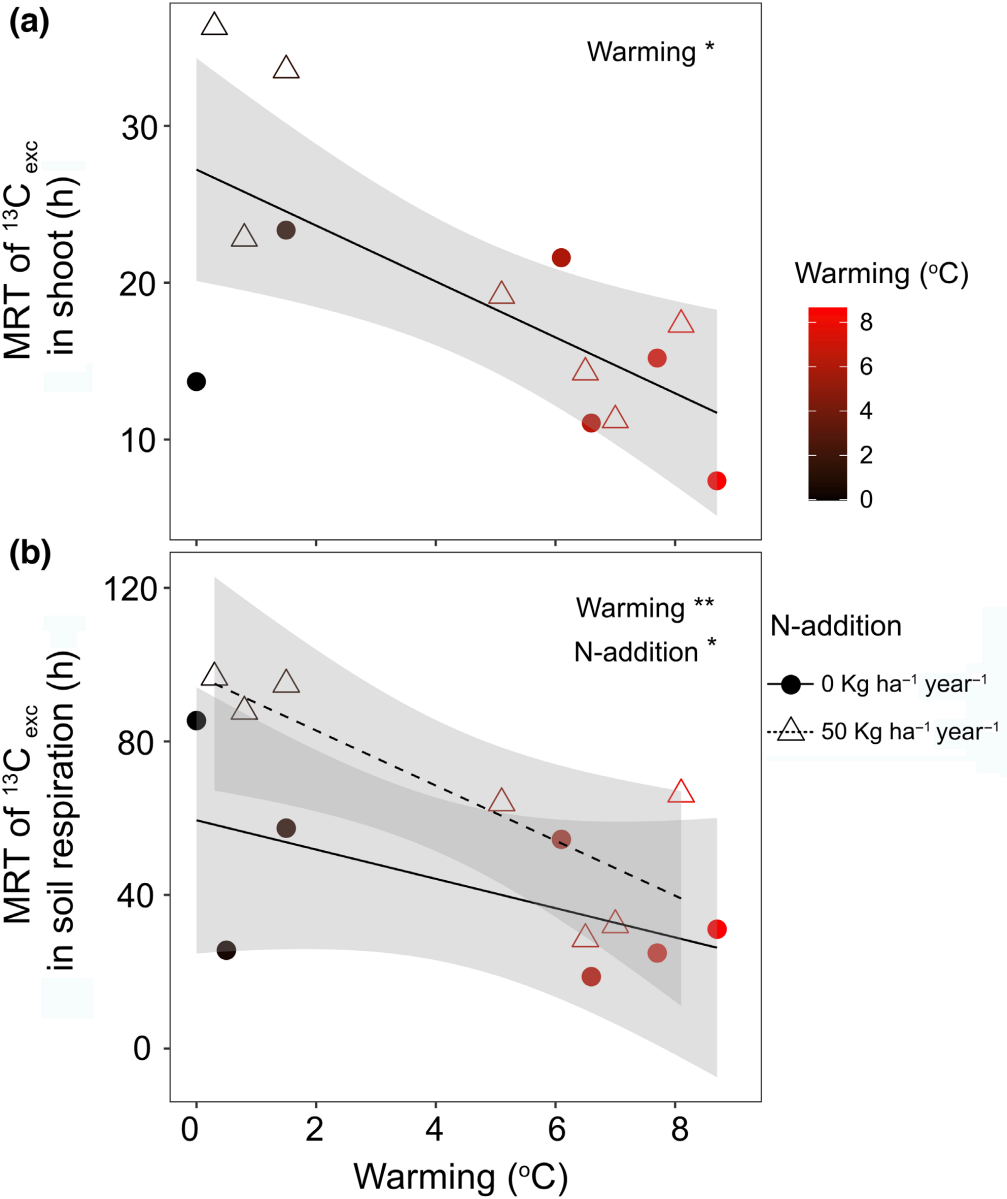
Mean residence time (MRT) of ^13^C excess in (a) shoot and (b) soil respiration in response to warming and N addition. Circles and triangles indicate measurements from unfertilized and fertilized (N‐addition) plots, respectively. The ^13^C excess in each plot was fitted with an exponential model (Equation 6). The MRT as calculated as the time required to reduce ^13^C excess to 1/e of its initial value. Asterisks denote significant warming and N addition treatment effects (*p* value: <.05 *; <.01 **).

In all plots, the absolute amount and relative (i.e., expressed per total tracer assimilated by the canopy) ^13^C excess in shoot biomass decreased exponentially after labelling (Figure 2a,b). Soil warming caused a more rapid decline in both the absolute and relative amount of ^13^C excess in shoots (Figure 2a,b; *t*‐value = −2.3, df = 50, *p* < .01), reducing the MRT of ^13^C (Figure 3a; *t*‐value = −2.9, df = 10 *p* < .05). N addition individually and in combination with warming did not significantly affect the ^13^C excess the MRT of ^13^C in shoots. In fine roots, the absolute and relative ^13^C excess peaked on day 1 after labeling and slowly declined afterwards. Warming decreased the ^13^C excess (both absolute and relative amount) in roots (*t*‐value = −4.3, df = 49, *p* < .001), N addition increased (*t*‐value = 2.3, df = 49, *p* < .05) the ^13^C excess (absolute and relative amount), whereas the interaction effect was not significant. Warming and N addition together did not affect the absolute and relative amount of ^13^C excess in soil EOC. Warming increased the absolute and relative ^13^C excess in MB (*t*‐value = 2.9, df = 50, *p* < .05) and SR (*t*‐value = 4.9, df = 63, *p* < .01). N addition individually and in combination with warming did not affect ^13^C excess in MB and SR. The ^13^C excess in SR declined exponentially after labelling. The MRT of ^13^C excess in SR decreased under warming and increased under N addition (Figure 3b; warming: *t*‐value = −3.2, df = 11, *p* < .01; N addition: *t*‐value = 2.2, df = 11, *p* < .05).

### 3.3 Testing of direct versus indirect effects of warming and N addition on carbon allocation

**FIGURE 4:**
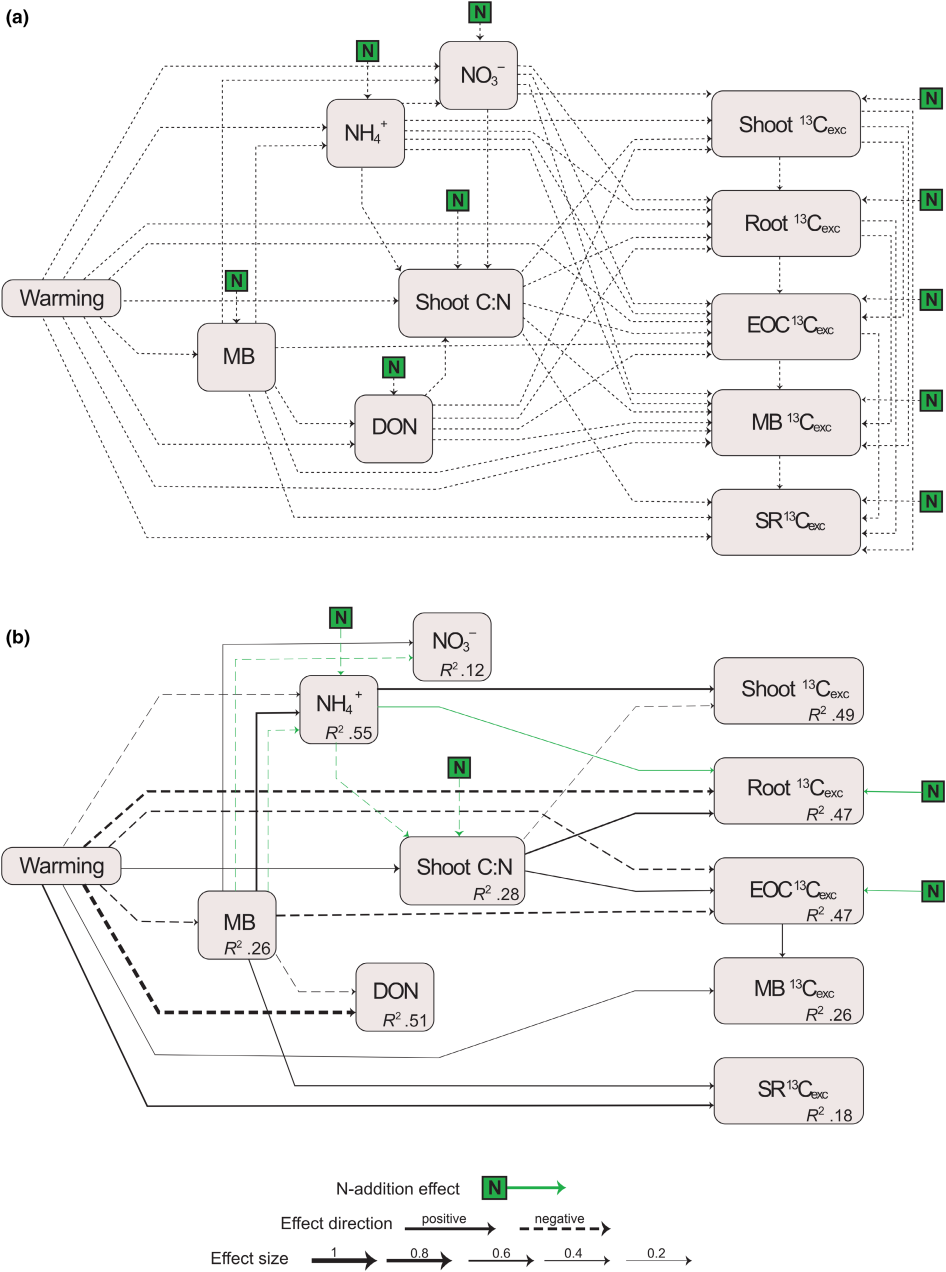
(a) Initial (hypothesized) and (b) final (retained) structural equation model testing the direct and indirect effects of warming on the relative amount of ^13^C excess in shoots, roots, extractable organic carbon (EOC), microbial biomass (MB), and soil respiration (SR). The green arrows represent the effects of N addition and its interaction effect. The thickness of the arrows represents the effect sizes (standardized path coefficients). Black solid and dashed arrows indicate significant positive and negative effects, respectively. Dotted arrows represent hypothesized effects.

We used structural equation modelling to test the direct and indirect effects of warming and N availability on the allocation of recently photosynthesized C (relative to initial label ^13^C incorporated; Figure 4a). We hypothesized that warming affects MB, which, in turn, affects soil organic and inorganic N availability and consequently the C:N ratio of plant biomass (Figure S6). We expected that at high soil N availability the allocation of recent C would be increased for shoot growth and decreased for belowground inputs.

Under warming, MB, DON and ${\mathrm{NH}}_{4}^{+}$ in soil was decreased and C:N in shoot biomass was increased (Figure 4; Figure S6). Decreased ^13^C _exc_ in shoots under warming was indirectly associated with decreased soil ${\mathrm{NH}}_{4}^{+}$ and increased C:N in shoot biomass. Warming strongly and directly decreased ^13^C _exc_ in roots and soil EOC, but indirectly increased ^13^C _exc_ in roots and EOC through increased C:N in shoot biomass. MB decreased ^13^C _exc_ in soil EOC and was linked to increased ^13^C _exc_ in MB. Lastly, warming and MB increased ^13^C_exc_ recovered in SR. Under N addition, ${\mathrm{NH}}_{4}^{+}$ was decreased in the soil, and was linked to decreased shoot C:N. N addition increased ^13^C _exc_ in roots and soil EOC. Moreover, increased ^13^C _exc_ in roots was indirectly associated with decreased ${\mathrm{NH}}_{4}^{+}$ under N addition. Thus, overall, the SEM shows that warming directly decreased the recent C in root and soil EOC, and increased the recent C in MB and SR. Indirectly, warming decreased plant and soil N availability and consequently decreased recent C in shoot and increased recent C in roots, and soil EOC. N addition increased N content in plants, ${\mathrm{NH}}_{4}^{+}$ was lower in soil, and the allocation of recent C was increased in roots and soil EOC.

### 3.4 Temporal dynamics of SR in relation to environmental drivers

**FIGURE 5:**
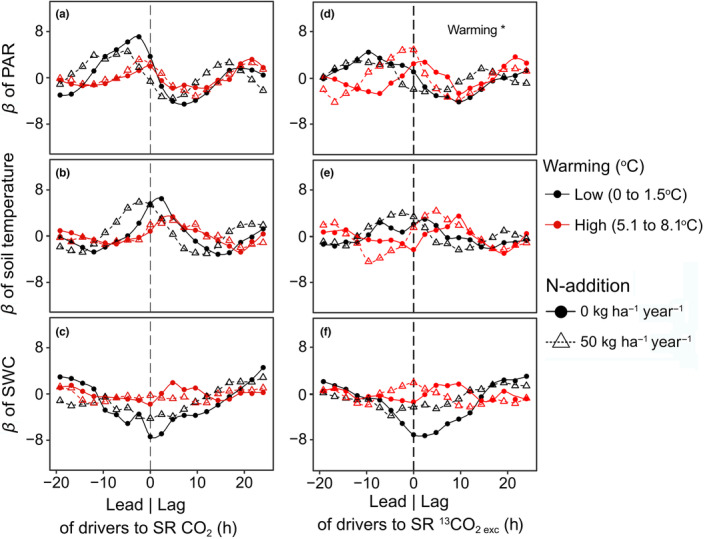
Cross correlation analysis of soil‐respired CO_2_ (total and ^13^CO_2_ from pulse labelling) and its environmental drivers under warming and N‐addition. The standardized regression coefficients of photosynthetically active radiation (PAR) (a, d), soil temperature (b, d) and soil water content (SWC; c, f) show the relationship of the drivers with soil‐respired CO_2_ and excess ^13^CO_2_ (^13^CO_2 exc_) in dependence of time lags between soil respiration (SR) and drivers. Negative values on the x‐axis indicate that driver fluctuations preceded fluctuations in SR and that therefore the drivers lead SR. Positive values indicate the opposite. Circles and triangles indicate measurements from unfertilized and fertilized (N‐addition) plots, respectively.

Environmental drivers such as soil temperature, SWC, and PAR (a proxy for photosynthesis) affected soil‐respired CO_2_ and ^13^CO_2 exc_ (Figure 5). We tested if warming or N additions altered the time‐lag between the diel dynamics of the drivers (PAR, soil temperature and SWC) and the response variables (soil‐respired CO_2_ and ^13^CO_2 exc_) using time‐series‐regression analysis. In unfertilized plots exposed to light warming (<1.5°C), dynamics of soil‐respired CO_2_ lagged 2.4 h behind PAR, and soil‐respired ^13^CO_2 exc_ lagged 9.6 h behind PAR (Figure 5a). In the unfertilized plots with stronger warming (5.1–8.7°C), the soil‐respired CO_2_ and ^13^CO_2_ did not lag, but were synchronized with variation in PAR (Figure 5a,b). Strong warming significantly decreased the time‐lag between the diel dynamics of PAR and soil‐respired ^13^CO_2 exc_ (Figure 5d). Under N addition, the lag of soil‐respired CO_2_ after PAR was 4.8 h in the lightly and 2.4 h in the more strongly warmed plots, and the lag of soil‐respired ^13^CO_2 exc_ was 2.4 h in the lightly warmed plots. Except on the lightly warmed N addition plots, the diel dynamics of soil temperature did not lead soil‐respired CO_2_ and ^13^CO_2 exc_ (Figure 5b,e). SWC did not display any diel variation (Figure S3).

## 4 DISCUSSION

Subarctic ecosystems hold significant amounts of C and are highly vulnerable to future warming. While it is well‐established that warming alters the heterotrophic pathway of C loss, warming effects on the autotrophic components of ecosystem C dynamics, especially the fate of recently photosynthesized C, are so far poorly understood. Here we studied how soil warming directly and indirectly altered C dynamics of a subarctic grassland. We found that 10 years of warming decreased GPP, increased belowground C allocation and SR, accelerated the belowground turnover of photosynthesized C, and turned the grassland into a major C source. N addition treatments suggest that while microbes were increasingly C limited with increasing degree of warming, plants were increasingly N‐limited, which constrained primary productivity.

### 4.1 Ecosystem productivity limited by N availability under warming

Warming and N addition significantly increased NDVI during the early season (Figure 1a). This suggests that warming advances the biological spring and causes early greening, which is consistent with other studies (Keenan & Richardson, 2015; Steltzer & Post, 2009) and previous observations at our study site (Leblans et al., 2017). Towards peak season, when NDVI in all plots reached saturation levels around 0.7, N addition increased NDVI. Towards the end of the growing season, NDVI was significantly decreased under warming, which suggests that warming also caused early senescence. Our result supports emerging findings of early senescence under long‐term warming (Keenan & Richardson, 2015; Wu et al., 2018), as has been previously shown for high‐latitude plant communities (Livensperger et al., 2019). Interestingly, at the same time NDVI was increased by N addition in interaction with warming, which indicates that the warming response of NDVI was N limited during senescence. While the interaction of warming and N addition on plant phenology has been poorly constrained (Shen et al., 2022) and has been shown to be independent in some case (Xia & Wan, 2013), our findings suggest that the effects of these factors may not be simply additive, but rather interact in complex ways. Moreover, our understanding of phenological responses to climate warming is currently limited to short‐term climate manipulation experiments (Piao et al., 2019). This is critical as the role of changing nutrient availability, which may gradually alter the original short‐term phenological responses, is not well understood (Leuzinger et al., 2011; Shen et al., 2022). Our study, which involved a 10‐year soil warming treatment, highlights the importance of considering N availability when studying the response of phenology to future warming and N addition. Since our study did not use a fully balanced random block design for the treatments it should be noted that the observed interactive effects of warming and N additions might be prone to some additional uncertainty. This highlights the importance of future studies exploring the potentially underrated implications of N availability for phenology in a warming world.

In our study, we also found indicators of N limitation under warming of ecosystem CO_2_ uptake during the peak growing season. Contrary to our hypothesis (H1), warming did not stimulate GPP (GPP_max_), while it increased ER (Figure 1c,d). The interaction of warming and N addition increased GPP_max_ (Figure 1c) and consequently increased net C uptake (Figure 1b). It has been previously suggested that warming would either decrease net C uptake by reducing SWC or would stimulate net C uptake under non‐water‐limited conditions (Quan et al., 2019) by increasing N availability in soil (Natali et al., 2012; Zhou et al., 2022). In our study site, SWC was greater than 35 vol.‐% in all the plots (Figure S2) and was not a limiting factor (Figure S3). Moreover, given that warming had a positive effect on GPP_max_ only when we experimentally added N, our study suggests that plant productivity was increasingly N‐limited under warming.

### 4.2 C allocation under warming

We hypothesized that warming would increase N availability in soil and in consequence increase recently photosynthesized C in shoots and decrease belowground C allocation (H2). Contrary to our hypothesis, warming decreased both the absolute and relative amount of recently photosynthesized C (^13^C _exc_) in shoots and roots (Figure 2a,b) indicating that more recent C was respired aboveground (Figure 1d) or allocated to soil (Figure 2a,b). Decreased shoot growth and increased belowground C allocation are typical responses to N limitation (Chen et al., 2018; Fellbaum et al., 2012; Gutknecht et al., 2012; Moreau et al., 2019). Our SEM‐based analysis suggests that decreased recent C in shoots was mainly associated with decreased soil ${\mathrm{NH}}_{4}^{+}$ and increased shoot C:N under warming (Figure 4b; Figure S6), which indicates plant N limitation. Moreover, the N addition experiment caused decreased shoot C: N, indicating that plant N uptake exceeded dilution by growth (Figure 4b; Figure S6).

In soil, warming increased the amount of recent C allocated to microbes (Figures 2 and 4b), though it had limited effects on microbial community composition and community size (Verbrigghe, Meeran, et al. 2022). Our results are in line with previous findings that SOC depletion upon warming can lead to C limitation of the microbial community (Verbrigghe, Leblans, et al. 2022; Walker et al., 2018), which makes them more dependent on labile plant C inputs in the rhizosphere. Our results from the N addition experiment also indicate that microbes were not N limited (Figure 2); although N additions increased plant tissue N concentrations (Figure 4b), productivity (Figure 1a–c) and belowground growth (Figures 2a,b and 4b), it did not alter the response of C allocation to microbes, which suggests that no plant‐mediated N addition effects occurred (Verbrigghe, Meeran, et al. 2022). Overall, our findings suggest that warming could tighten the plant‐microbial coupling through increased transfer of C highlighting the importance of understanding the interaction between N and C demands of plants and microbes (Čapek et al., 2018; Soong, Fuchslueger, et al., 2020).

### 4.3 Accelerated turnover and release of C under warming

In our study, warming increased total SR rates (Figure 1e), as suggested by previous studies and a meta‐analysis (Carey et al., 2016; Wang et al., 2019). It has previously also been shown that a warming‐induced decrease of SWC can override the direct positive effects of warming (Fang et al., 2018; Yan et al., 2021). In our study on a subarctic grassland with high and evenly distributed annual precipitation (Sigurdsson et al., 2016), SWC was not a limiting factor also in warmed plots (Figure S3). In previous studies that report increased SR under warming, the source of increased SR has been suggested to be primarily soil organic matter turned over by heterotrophic soil microbial activity (Graham et al., 2014; Schindlbacher et al., 2009; Wang et al., 2017). Our results show that also the autotrophic source of SR can be increased under warming, as the amount of recently photosynthesized C in SR increased (Figure 2b). While we found a significant linear overall relationship between warming and SR, there was a major increase in SR at a threshold around ~6°C warming, beyond which a small increase in warming led to a strong increase in SR (Figure 1e). This finding is consistent with the concept of a temperature sensitivity threshold, beyond which SR becomes more sensitive to temperature changes, resulting in a non‐linear relationship (Luo & Zhou, 2006). A broader overreaction of the studied subarctic grassland to warming was observed already after 5–8 years of warming, and has been suggested to be likely due to physiological adjustments of soil organisms (Walker et al., 2020). Our study shows that an increased temperature sensitivity of SR was sustained also after 10 years, and that warming not only increased allocation of recent C to SR, but also the turnover of recently photosynthesized C. This was indicated by decreased MRT of recent C in shoots and SR (Figure 3) and a decreased lag of photosynthesis and soil‐respired ^13^CO_2 exc_ (Figure 5a,d). Our findings thus confirm hypothesis (H3) that warming would increase the turnover of recently photosynthesized carbon and increase SR. Our study thus not only supports the notion that GPP and SR are tightly coupled (Bahn et al., 2009; Kuzyakov & Gavrichkova, 2010) and that therefore photosynthesis exerts an important control on SR (Han et al., 2014; Meeran et al., 2021; Vargas et al., 2011), but also suggests that this coupling was strengthened and accelerated by warming.

**FIGURE 6:**
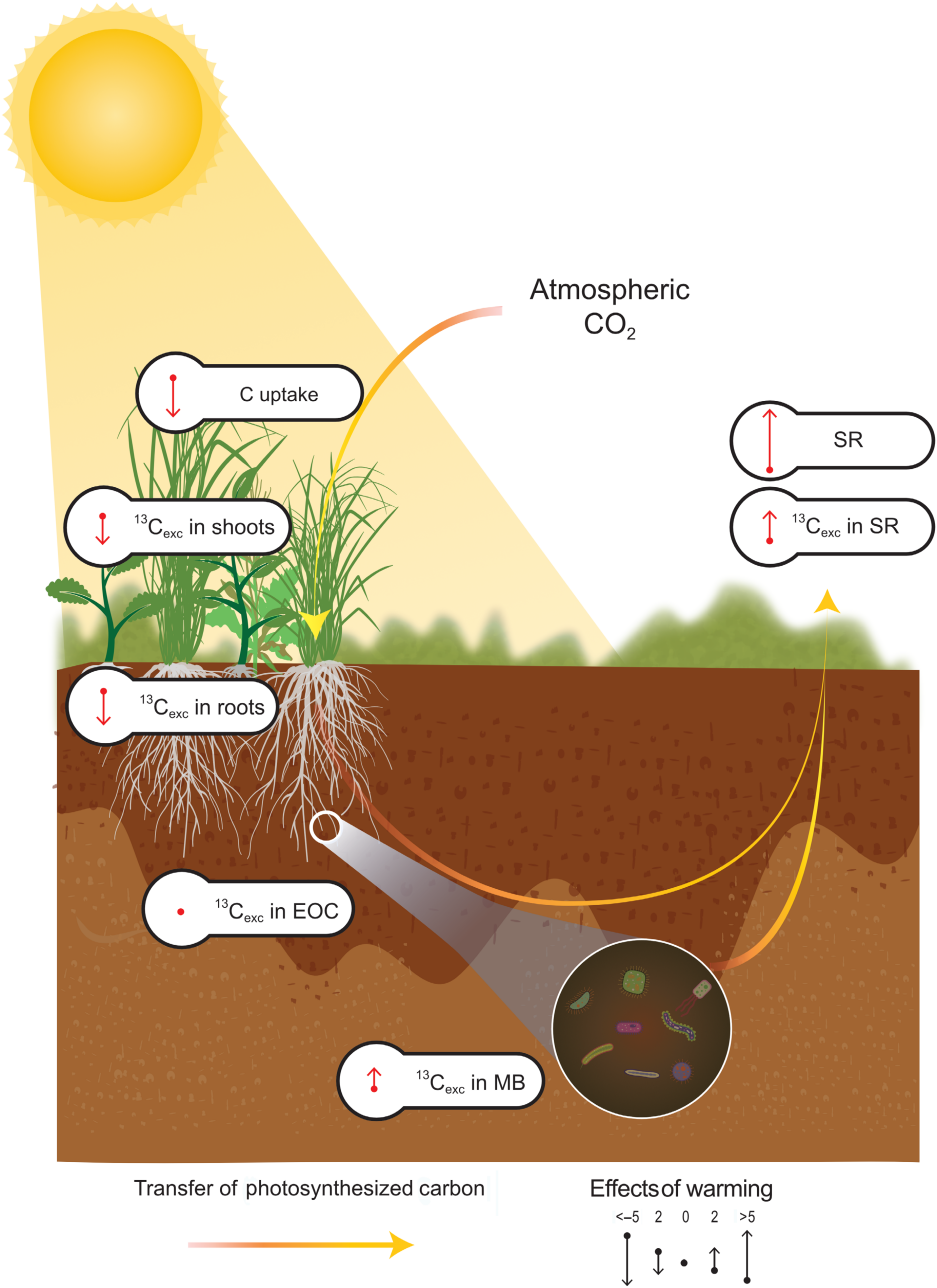
Graphical summary of the effects of 10 years of soil warming on the transfer of C from net CO_2_ uptake to soil respiration (SR). The warming effect sizes on excess ^13^C (^13^C_exc_) in shoot, root, soil extractable organic carbon (EOC), microbial biomass (MB) and SR are standardized regression coefficients obtained from linear mixed effects models. The upward and downward pointing arrows represent increasing and decreasing effects of warming, respectively.

We had also hypothesized that N addition would diminish the coupling of GPP and SR under warming (H3), because N addition could increase allocation to aboveground plant growth and thus decrease the belowground turnover and increase the residence time of recent C (Xiao et al., 2019). In our study, N addition indeed significantly increased the MRT of recent C in SR (Figure 3b) and therefore reduced the soil warming effects on belowground turnover. While photosynthetic C uptake was limited by N, increased belowground allocation and accelerated C release indicate that higher proportion of recently photosynthesized C could be lost from subarctic grassland under future warming (Figure 6). Previous research from our study site showed that in response to warming soil organic carbon stocks decreased by a 9.1 ± 2.1% °C^−1^ during the first 5 years and then stabilized (Verbrigghe, Leblans, et al. 2022). These dynamics have been suggested to be related to changes in MB and its activity (Walker et al., 2018). Our findings indicate that the grassland's capacity to offset warming‐induced heterotrophic C loss may be limited by limited C uptake and increased belowground turnover and SR.

It should be noted that the pulse labelling experiment was conducted during the peak period for C uptake and allocation, when the effects of warming and N availability were expected to be most pronounced. While it can be assumed that this period therefore also had the strongest imprint on belowground C allocation and thus on the coupling of GPP and SR, it is unknown whether similar individual and combined effects of the imposed global change treatments would occur also during spring or fall, considering the strong seasonality of C cycle processes. As discussed above, warming altered the dynamics of NDVI in spring and fall, and the warming response of NDVI was N limited during senescence (Figure 1a), supporting the notion of seasonally variable effects. Thus, to obtain a more comprehensive understanding of the responses of C cycle processes to direct and indirect warming effects on an annual scale, future studies should account for such possible seasonal shifts in CO_2_ fluxes and allocation processes.

## 5 CONCLUSION

From our study, we conclude that a decade of soil warming significantly altered photosynthetic C uptake, allocation, and turnover in a subarctic grassland. Under soil warming (1) GPP was N limited; (2) allocation of recently photosynthesized C from shoots to roots was decreased and C allocation to MB was increased; and (3) the turnover of recently photosynthesized C in soil was accelerated, causing faster release of recently assimilated C from ecosystem to the atmosphere, and leading to a net C loss from the grassland. Unexpectedly, 10 years of soil warming reduced N availability for plants and thus reduced net C uptake and allocation to shoots, while increasing belowground C allocation, turnover rates, and SR. Our study highlights the importance of belowground C allocation and C‐N interactions for understanding and predicting C dynamics of subarctic ecosystems in a warmer world.

## AUTHOR CONTRIBUTIONS

The study was designed by Michael Bahn, Jennifer L. Soong, Sara Vicca and Ivan Janssens, field work was performed by Kathiravan Meeran, Niel Verbrigghe, Johannes Ingrisch, Lucia Fuchslueger, Lena Müller, Ivan Janssens and Michael Bahn, Bjarni D. Sigurdsson and Páll Sigurðsson operated the field site, carbon isotope analyses in the lab were conducted by Margarete Watzka, Herbert Wachter designed and constructed the chamber for gas flux measurements and labeling, Kathiravan Meeran and Niel Verbrigghe analyzed the data, and Kathiravan Meeran and Michael Bahn wrote the manuscript, with feedbacks and inputs from Johannes Ingrisch and all co‐authors.

## CONFLICT OF INTEREST STATEMENT

The authors declare that they have no known competing financial interests or personal relationships that could have appeared to influence the work reported in this paper.

## Supporting information


Appendix S1.

## Data Availability

The data that support the findings of this study are openly available in Zenodo repository at https://doi.org/10.5281/zenodo.8026985.
