# Supplementary material for: Individual and interactive effects of warming and nitrogen supply on CO2 fluxes and carbon allocation in subarctic grassland
Source: Glob Chang Biol. 2023 Jul 10;29(18):5276–91. doi: 10.1111/gcb.16851 (PMC10962691; doi:10.1111/gcb.16851)
Supplement: Supplementary file 1 — Appendix S1. [file GCB-29-5276-s001.docx]

**Supporting information**


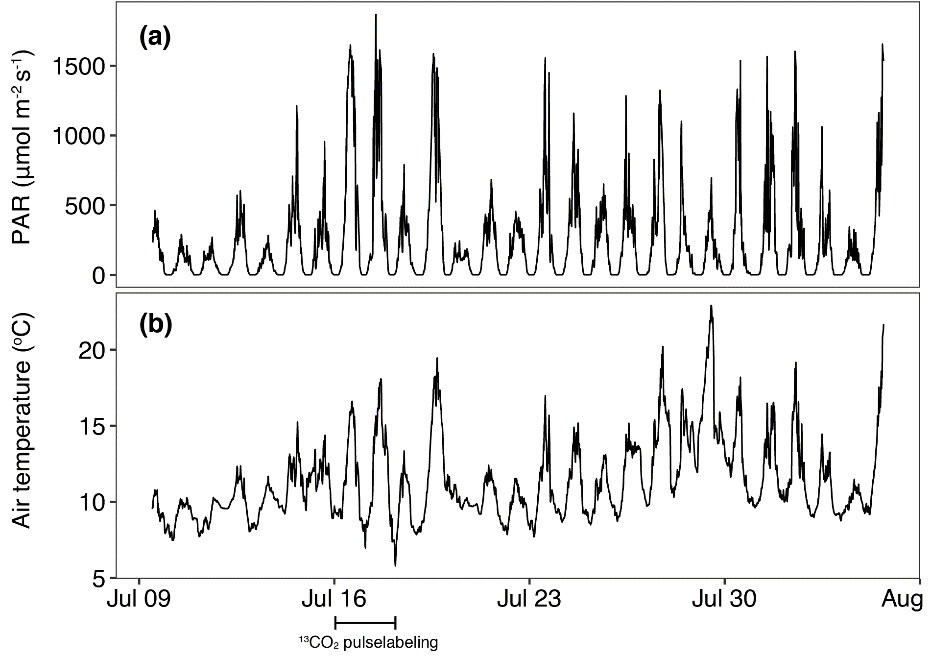


**Figure S1:** Photosynthetic active radiation (PAR) and air temperature at the study site measured during the main study period.


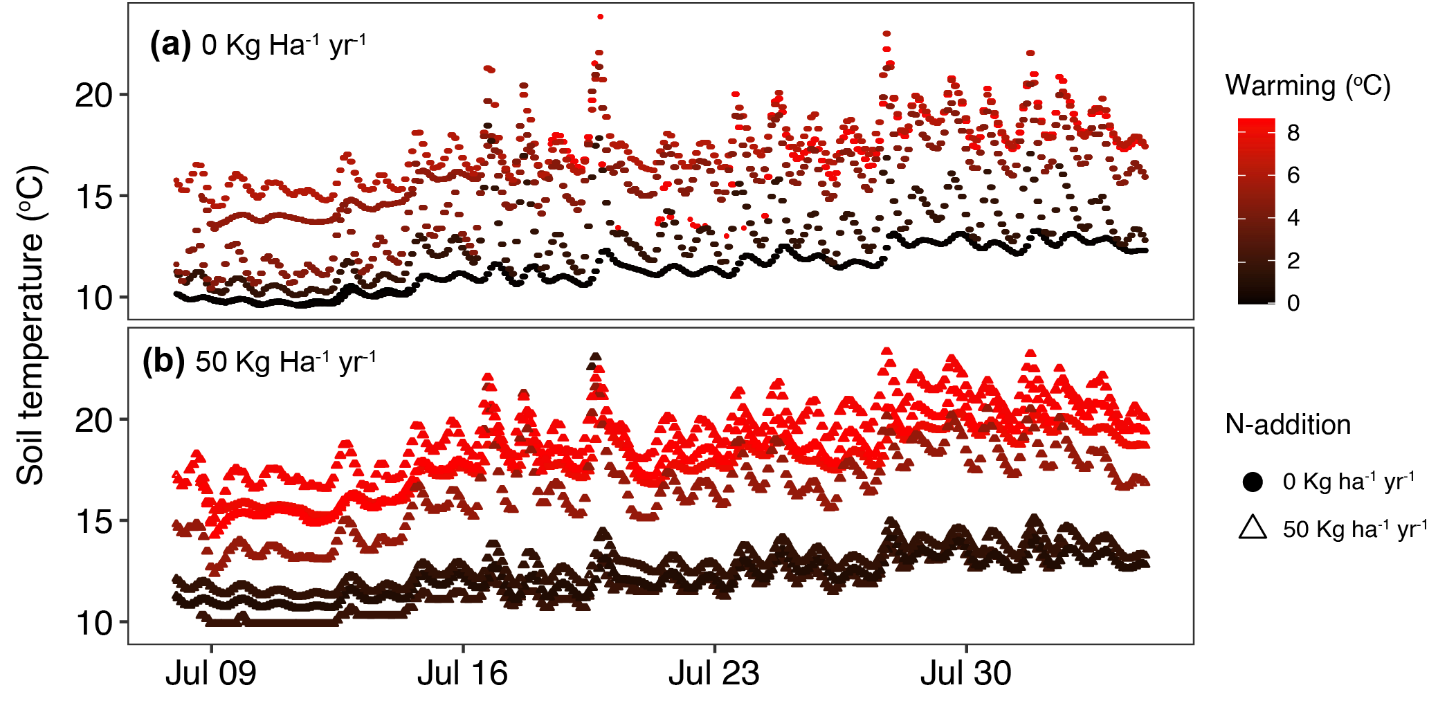


**Figure S2**: Hourly means of soil temperature measured at 5 cm soil depth during the main study period.


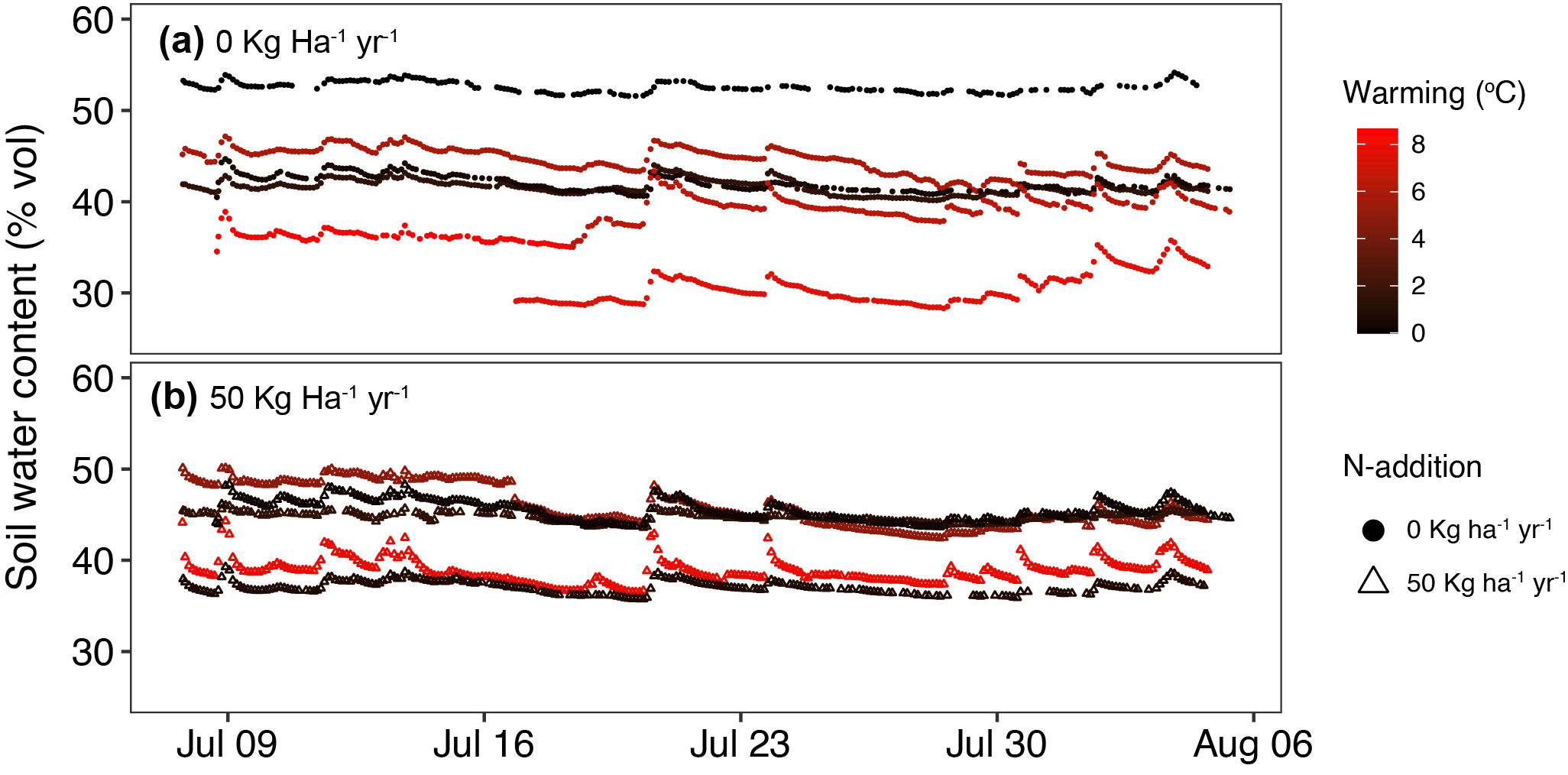


**Figure S3**: Hourly means of soil water content measured at 5 cm soil depth during the main study period.


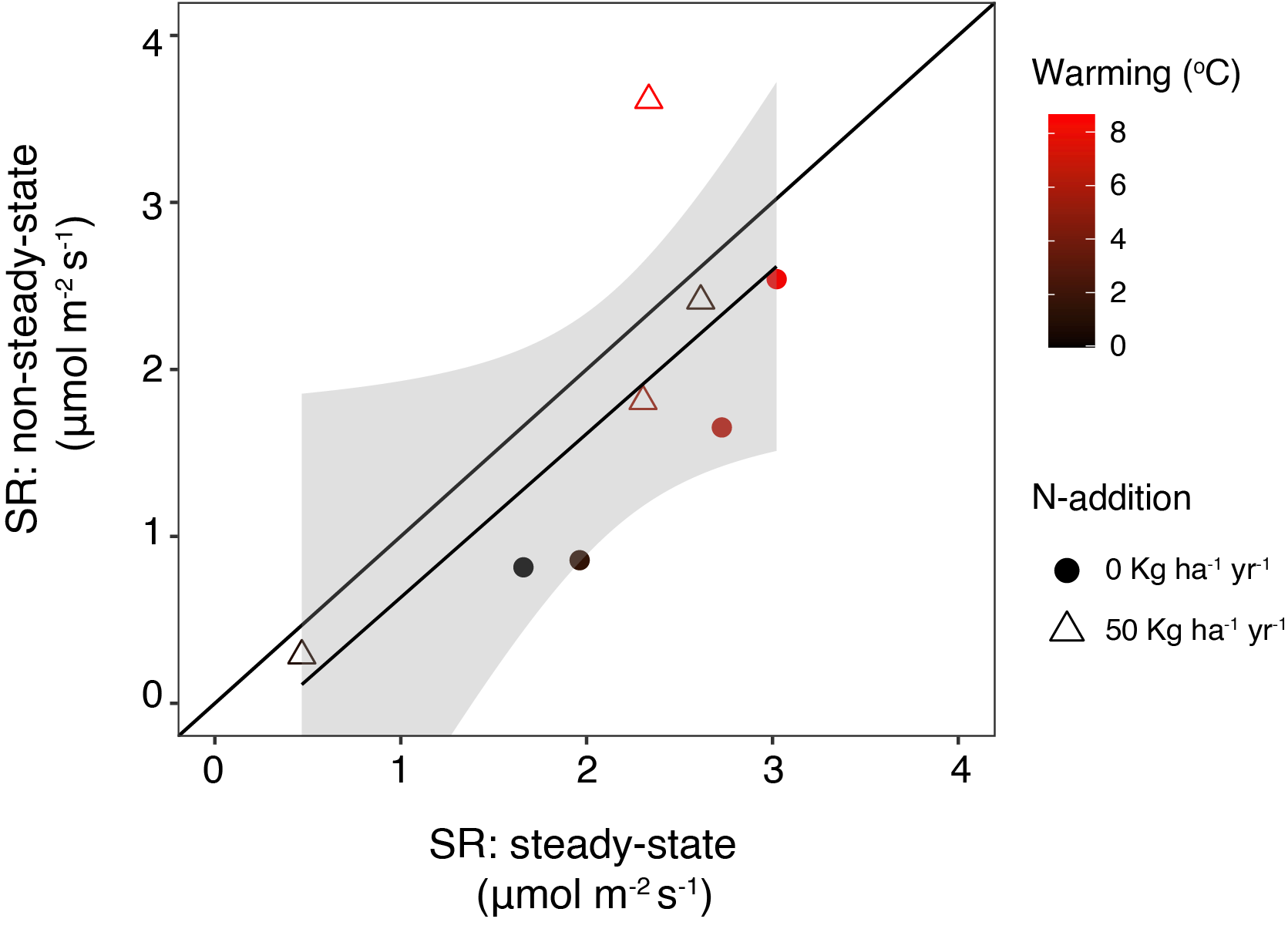


**Figure S4**: Comparison of steady-state and non-steady-state soil respiration (SR) measurements. Non-steady-state measurements were made on eight plots where also steady-state measurements were performed. The non-steady state measurements were made between 9 AM to 4 PM on July 30, 2018. The mean of steady-state SR measured during the same time duration was used for comparison.


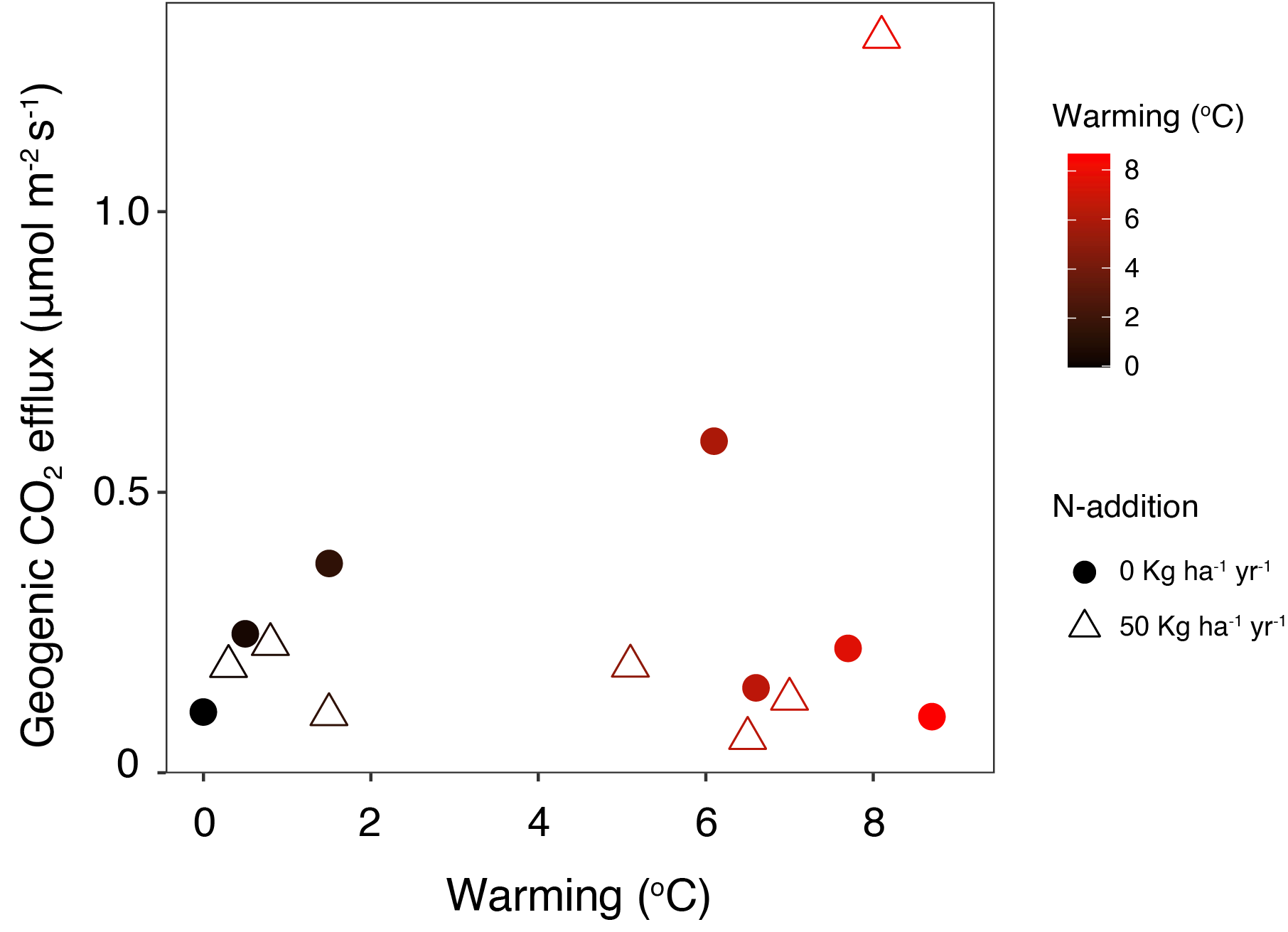


**Figure S5:**  Geogenic CO_2_ efflux estimated using a two-pool isotope mixing model on soil CO_2_ efflux. Warming did not consistently affect geogenic CO_2_ efflux.


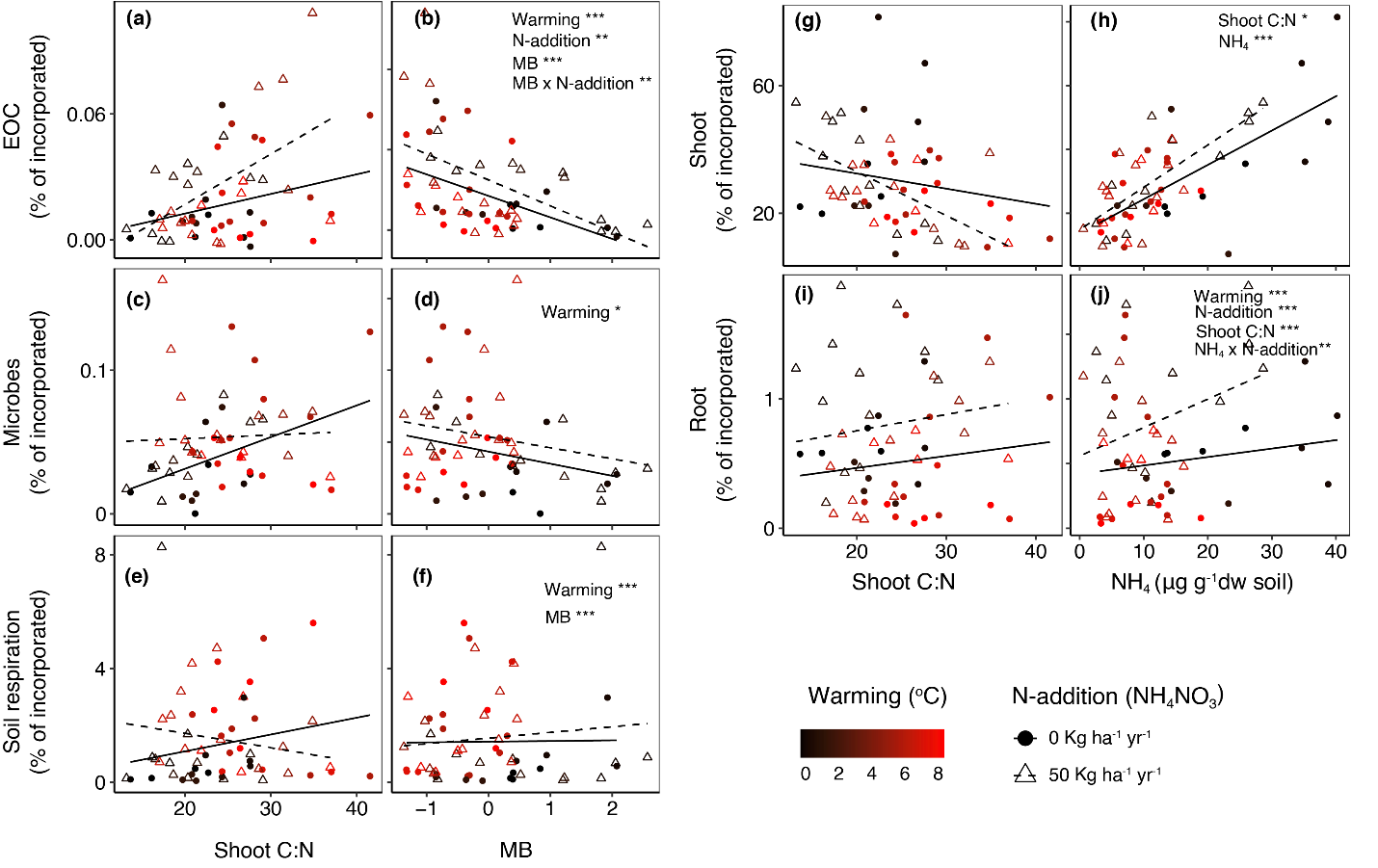


**Figure S6:** Effects of warming, N addition and microbial biomass and shoot C:N on the relative ^13^C excess (i.e. the proportion of ^13^C taken up during pulse labelling) in (a,b) EOC, (c,d) microbes, (e,f) soil respiration, (g,h) shoot and (I,j) root. MB- Microbial biomass.
